# Radiology blues: Comparing occupational blue-light exposure to recommended safety standards

**DOI:** 10.4102/sajr.v27i1.2522

**Published:** 2023-01-31

**Authors:** Mari Wentzel, Jacques Janse van Rensburg, Jacobus J. Terblans

**Affiliations:** 1 Department of Clinical Imaging Science, Faculty of Health Sciences, University of the Free State, Bloemfontein, South Africa; 2 Department of Physics, Faculty of Natural and Agricultural Sciences, University of the Free State, Bloemfontein, South Africa

**Keywords:** blue-light hazard, ocular health radiology, blue-light radiance, occupational blue-light hazard, blue-light exposure, safe occupational exposure

## Abstract

**Background:**

The blue-light hazard is a well-documented entity addressing the detrimental health effects of high-energy visible light photons in the range of 305 nm – 450 nm. Radiologists spend long hours in front of multiple light-emitting diode (LED)–based diagnostic monitors emitting blue light, predisposing them to potentially higher blue-light dosages than other health professionals.

**Objectives:**

The authors aimed to quantify the blue light that radiology registrars are exposed to in daily viewing of diagnostic monitors and compared this with international occupational safety standards.

**Method:**

A limited cross-sectional observational study was conducted. Four radiology registrars at two academic hospitals in Bloemfontein from 01 October 2021 to 30 November 2021 participated. Diagnostic monitor viewing times on a standard workday were determined. Different image modalities obtained from 01 June 2019 to 30 November 2019 were assessed, and blue-light radiance was determined using a spectroscope and image analysis software. Blue-light radiance values were compared with international safety standards.

**Results:**

Radiology registrars spent on average 380 min in front of a diagnostic display unit daily. Blue-light radiance from diagnostic monitors was elevated in higher-intensity images such as chest radiographs and lower for darker images like MRI brain studies. The total blue-light radiance from diagnostic display units was more than 10 000 times below the recommended threshold value for blue-light exposure.

**Conclusion:**

Blue-light radiance from diagnostic displays measured well below the recommended values for occupational safety. Hence, blue-light exposure from diagnostic monitors does not significantly add to the occupational health burden of radiologists.

**Contribution:**

Despite spending long hours in front of diagnostic monitors, radiologists’ exposure to effective blue-light radiance from monitors was far below hazardous values. This suggests that blue-light exposure from diagnostic monitors does not increase the occupational health burden of radiologists.

## Introduction

Blue light and its relation to health swiftly became one of the buzzwords in a myriad of discussions on physical condition and well-being.^1^ Detrimental health outcomes linked to increased screen time include a wide range of ocular effects, influence on hormone secretion (specifically melatonin and adrenocorticotrophic hormone [ACTH]), mental health illnesses, musculoskeletal ailments and skin conditions.^2,3,4,5^

As a result of the fundamental nature of their work, radiologists spend long hours scrutinising images on visual display units (VDUs) – often up to five brightly illuminated monitors at a time. In addition to this, smartphones, tablets, personal computers and televisions for purposes of communication, referencing, administrative tasks at work, studying and leisure also add a significant component to the total daily screen time.^6^

An important contributing factor to the unfavourable effect of prolonged screen time is greater exposure to the short wavelength photons at the ultraviolet end of the visible light spectrum or blue light.^6^ Increased exposure to VDUs and other electronic devices potentially places the radiologist in a higher-risk group to experience negative outcomes related to blue light. It was previously demonstrated that eye strain among healthcare professionals is common but meaningfully increased amongst radiologists when compared with other specialists such as paediatricians.^7^ In the current era of digital radiology, where most duties are performed using electronic picture archiving and communication system (PACS) technology on computers and VDUs, an increased prevalence of ocular health matters was identified among radiologists.^8^ This may lead to a temporary compromise in working and radiological diagnostic efficiency and ultimately individual visual acuity loss.^9^ From an occupational health perspective, the preservation of visual acuity of a radiologist should be considered a crucial concern.^10^ In order to obtain this goal, a good understanding of the contributing parameters and especially the ocular effects of blue light exposure when working on VDUs is important.

Chronic eye strain and the subsequent symptoms that follow and affect ocular health and vision, as well as mental health illness and burnout, especially during residency or registrar periods, were identified as occupational hazards radiologists face that are unrelated to radiation.^11^ Burnout among registrars occurs commonly. Research has shown intricate relationships between mood states, mental health and exposure to blue light; hence, blue-light exposure may add to the mental health burden.^12,13^

The concept of potential phototoxic effects to the retina related to blue-light exposure is referred to as the blue-light hazard. Blue light imparts retinal phototoxicity via energy deposition with direct subsequent damage to photoreceptor cells.^14^ The International Commission on Illumination (CIE, derived from its French title, the Commission Internationale de l´Eclairage) released a statement on the blue-light hazard in 2019, which specified that light sources used in general lighting and similar applications are not likely to pose any risk.^15^

In this study, the blue-light exposure from VDUs of radiology registrars working in a training institution was quantified and compared with recommended international safety standards. By analysing this parameter, insight was gained into one of the main occupational health challenges in radiology.

### Quantifying blue light

The American Conference of Governmental Industrial Hygienists (ACGIH^®^) have developed a series of Threshold Limit Values (TLVs^®^) and Biological Exposure Indices (BEIs^®^) to serve as guidelines for safe occupational exposure to chemical substances and physical agents, including non-ionising radiation such as blue light.^16^ These values, in conjunction with the recommendations from the International Commission on Non-Ionising Radiation Protection (ICNIRP) were used as directives for comparison in this study.^17^

The radiant luminance of blue light, *L_B_*, from the source in question is used to assess the amount of light incident on the retina. Radiant luminance is a radiometric concept deduced from radiant flux, flux density and intensity. Radiant flux, *Φ_e_*, is an indication of the amount of energy transmitted for a specific time period and is measured as energy units transferred per unit of time, or Watts (J/s). Flux density, €, is obtained when radiant flux is measured in a specific area unit. In the case of ocular photometry, it is the radiant flux at the retina (W/cm^2^).

**FIGURE 1:**
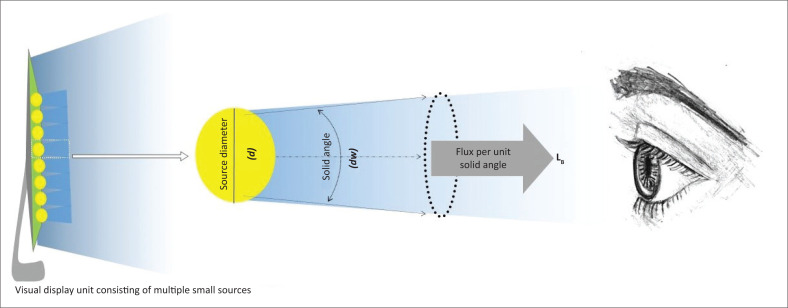
An area of the visual display unit is enlarged. The light emission occurs in a diffuse, three-dimensional distribution. When considering the flux per unit solid angle and diameter, the radiant luminance, *L*_*B*_, incident on the eye of the observer can be determined.

Monitors of VDUs consist of multiple arrays of light-emitting diode (LED) light sources emitting light in a Lambertian distribution. When looking at a monitor of a VDU, the sum of all the small sources combined is emitted in a diffuse manner, as illustrated. Light emitted from VDUs is considered to be originating from an extended source. Emission occurs from the surface in all directions, hence the solid angle subtending the source must be considered to be accurate and include all light emissions (Figure 1).^18^

The effective blue-light radiance, *L*_*B*_, is the unit of significance when evaluating radiance dose to the retina. It is attained by summating or integrating the product of the radiance of the source for every wavelength and the blue-light hazard weighting function B(*λ*):^16,17^


$$L_{B}=\sum_{305}^{700}L_{\lambda }\times \text{B}(\lambda )\times \Delta \lambda (\text{W}/cm^{2}\text{sr})$$ [Eqn 1]

The blue-light hazard weighting function represents the relative sensitivity of the human eye and the potential of specific wavelengths in the blue-light range to induce photochemical injury. In the case of an aphakic eye, a hazard function, A(*λ*), with heavier weighting is used to compensate for the absence of the lens.^17^

When assessing typical workday exposures, viewing times will exceed 167 min (10 000 s), and the exposure limit of the blue light–weighted effective radiance *L_B_* is defined by the ICNIRP as:^13^


$$L_{B}\leq 0.01W/cm^{2}sr$$ [Eqn 2]

## Methodology

A cross-sectional observational study was conducted to establish blue-light exposure. Participants consisted of four radiology registrars working at two academic hospitals in central South Africa from 01 October 2021 to 30 November 2021. Measurements were performed for a total duration of 20 working days.

Only the registrars working at CT and MR imaging stations were included. Reporting of daily scheduled CT and MR scans involves the prolonged evaluation of images on a minimum of three brightly lit computer monitors, which include two dedicated diagnostic display (DDD) units and one standard monitor. Other workstations such as ultrasound, mammography, fluoroscopy and interventional radiology are less predictable than CT and MR, as they involve more active and procedural patient interaction. These stations automatically yield less screen time and subsequent blue-light exposure and were excluded. During conduction of this study, the department did not have a registrar allocated to full-time plain film reporting; hence, it was not possible to include a participant reporting only chest radiography (CXR).

**FIGURE 2:**
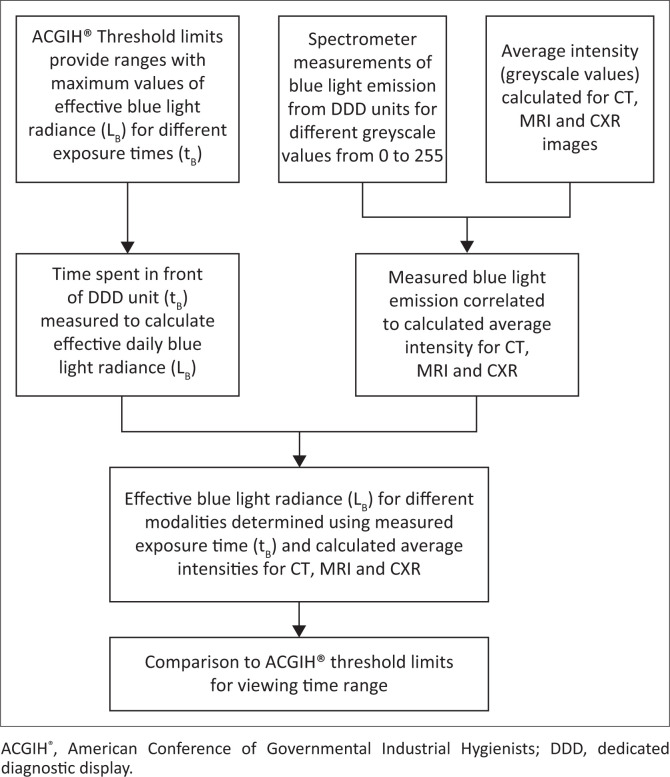
The method used to determine blue-light radiance consisted of two parallel parts. Registrar viewing time was measured and applied to determine acceptable threshold limits. Spectral data measured for different greyscale values were used to calculate effective blue-light radiance. Average image intensities for modalities were linked to greyscale values, providing a relation to deduce effective blue-light radiance.

This study encompassed two pathways that were ultimately combined to obtain effective blue-light radiation (Figure 2). Current photometric systems used to evaluate blue light have restricted practical capabilities outside laboratory settings because of factors such as weight, bulky size, expensive components and difficulty in providing reproducible accuracy.^19^ An experimental setup to measure continuous, cumulative blue-light exposure from the whole surface of a DDD is not feasible in a practical diagnostic radiology setup. The authors relied on theoretical principles to deduce the values of interest from a range of spectroscopic greyscale measurements from DDD units, measured registrar viewing times and calculations of intensity values of images (Figure 2).

### Registrar viewing times

The TLVs^®^ as determined by the ACGIH® are provided as maximum values for different viewing time ranges (Table 1).^16^ Daily viewing times were measured to determine the TLVs^®^ to be applied.

**TABLE 1:** Accepted exposure limits for blue light according to the American Conference of Governmental Industrial Hygienists (ACGIH®), effective blue-light radiance, *L_B_*, measured in (W/cm^2^sr), and time, *t_B_*, measured in seconds.

Category	Viewing duration	Recommended threshold limit
1	*L_B_* When viewing durations *t_B_* are less than 167 min, or approximately 2.8 h per day	$L_{B}\leq \frac{100}{t_{B}}$
2	Maximum acceptable duration of exposure *t_Bmax_* when *L_B_* exceeds 0.01 W/cm^2^sr	$t_{B\max}=\frac{100}{L_{B}}$
3	Acceptable exposure *L_B_* when viewing durations *t_B_* are greater than 167 min, or approximately 2.8 h per day	*L_B_* ≤ 0.01

Note: Time values indicated in minutes for ease of interpretation.

*Source:* The American Conference of Governmental Industrial Hygienists. ACGIH® Threshold Limit Values (TLVs) and Biological Exposure Indices (BEIs). Signature Publications, 2012; p. 871–1130

Participants logged the time they spent viewing images on DDD units by means of a stopwatch application. They activated the stopwatch whenever viewing was started and stopped it when they left the workstation or stopped viewing images on the DDD unit. Viewing times were recorded over the duration of 20 workdays to obtain an estimate of the total viewing time registrars spend in front of the DDD units per day.

### Instrumentation

The authors assessed a 3-MP diagnostic reporting monitor that was calibrated as per requirements for licence holders by the Department of Health in South Africa.^20^ Regular quality control tests were performed on these monitors by an accredited inspection body, and all results were within mandatory limits when the study was performed.^21^

**FIGURE 3:**
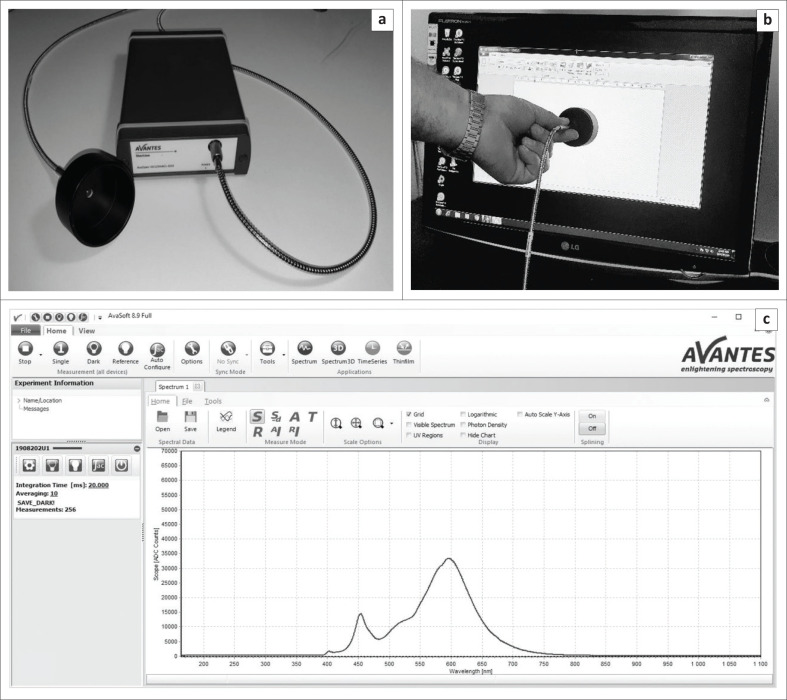
The spectroscope used for measurement, with attached plastic envelope to ensure reproducibility of measurements (a). Indicated in image (b) is a representation of how measurements were obtained. A screenshot from the proprietary software package accompanying the Avantes spectrometer demonstrates the curve, with spectral data obtained from the spectroscope indicating peaks at different wavelengths (c).

The AvaSpec-ULS2048CL Spectrometer, with optimal efficiency for non-ionising radiation in the visible range, was used for measurements of blue-light emission from DDD units. This spectrometer uses a fibre optic system to measure light emission from a source by counting the number of photons for each wavelength in a specific range. The fibre optic detector was mounted in a fixed plastic envelope to ensure reproducibility of all measurements in terms of area of measurement, influence of ambient light and distance of detector from monitor. The measured data were delivered in spectral form for different wavelengths of light, displayed as the number of photons of each wavelength on a graph (Figure 3). The spectrometer was calibrated to a standard source of white light using the principle of blackbody radiation and an incandescent tungsten lamp, emitting white light at a specific known temperature.^22^

### Establishing average intensities of different imaging modalities

**FIGURE 4:**
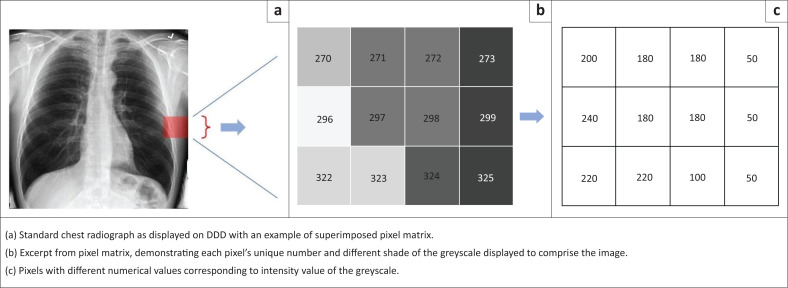
The pixel matrix that forms an image. Part (a) is a standard chest radiograph, with (b) a representation of an excerpt of the pixel matrix in the highlighted area. The numbers represent the different pixel ‘addresses’, and the different shades of grey correspond to the displayed image elements. Part (c) demonstrates the greyscale values displayed in each pixel. Darker pixels have lower values and brighter pixels higher values.

A radiological image displayed on a DDD unit comprises a matrix of different pixels. Each pixel is allocated a number that gives it an ‘address’ in the matrix and displays a certain value of the greyscale to make up the final image. The greyscale is a range of different intensities varying from black to white. Black is defined as no intensity and white is the highest intensity. A value of ‘0’ is assigned to black, and ‘255’ to white. A computer interprets an image as a range of different values allocated to each pixel in the matrix. Each pixel displays a specific value of the greyscale to form the image (Figure 4). These pixel values that make up an image can be displayed in a greyscale histogram, which is a graph that displays the number of pixels as a function for each intensity value of the greyscale.^22^

**FIGURE 5:**
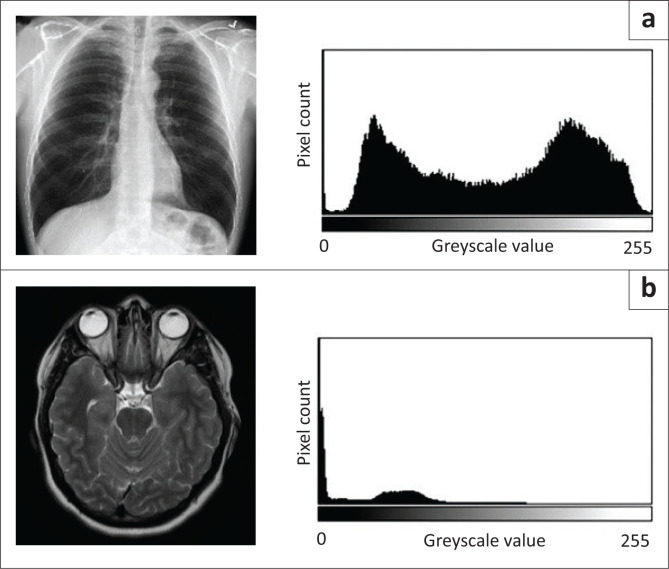
Histogram of a normal chest radiograph (a) and a single MR Brain T2 slice at the level of the brain stem (b), demonstrating the relative left shift of pixel greyscale values in the darker image.

An example of the histogram for the chest radiograph in Figure 4 is demonstrated in Figure 5. On the *x*-axis, greyscale values from 0 to 255 are presented, with the *y*-axis representing the number of pixels displaying each greyscale value. Also demonstrated in Figure 5 is a histogram of a single slice of an MR image. Intuitively, the MR image demonstrates larger amounts of darker image elements, explaining the relative shift of the histogram towards the left-sided, darker end of the greyscale. This observation is based on the standard presentation presets of the PACS system in the department under study and without adjusting any window settings. The bright chest radiograph, with greyscale values in a wide range, demonstrates a broad histogram with large amounts of different intensity values. If the intensity values of all the pixels in the histogram are summated, an average number, *I_avg_*, indicating the intensity of the image as a whole, can be determined.

Viewed content will play an important part in the dosage of blue light, as images with increased intensity will yield more bright light from the screen and, as a result, increased associated blue-range photons. For the purpose of this study, standard CXR, 5 mm axial slices of pre-contrasted CT images of the brain and 4 mm axial T1 and T2 pre-contrasted MR images of the brain were utilised. Commonly used modalities were selected to provide an indication of how looking at different imaging modalities may influence blue-light exposure.

The sample of assessed images was randomly selected and included 100 PA view chest radiographs, 30 pre-contrasted CT brain studies consisting of 5 mm slices and 25 pre-contrasted MRI brain studies, T1 and T2 sequences, 4 mm slices. Images were selected from all the studies performed at Universitas Academic Hospital from June 2019 to November 2019. The authors purposely chose a 6-month period that fell outside the South African national lockdown (announced at the end of March 2020) as the lockdown regulations and practices significantly reduced patient numbers in the months following their implementation. Arbitrary holiday periods of December and April were excluded to give a realistic representation of a standard workday. A list of total studies for the respective modalities during said time periods was obtained and entered into Microsoft Excel. Every study was allocated a number, and from this, random samples were selected.

Images from the selected studies were assessed using ImageJ, a public domain Java image processing software package.^23^ Average intensity values for a chest radiograph, CT brain and MRI brain were obtained. The average intensity value of an image correlates with a specific greyscale value. A range of greyscale values was then displayed on the DDD and spectroscopic measurements were performed. Resultant spectral curves were used to determine the blue-light radiance for each greyscale value.

**FIGURE 6:**
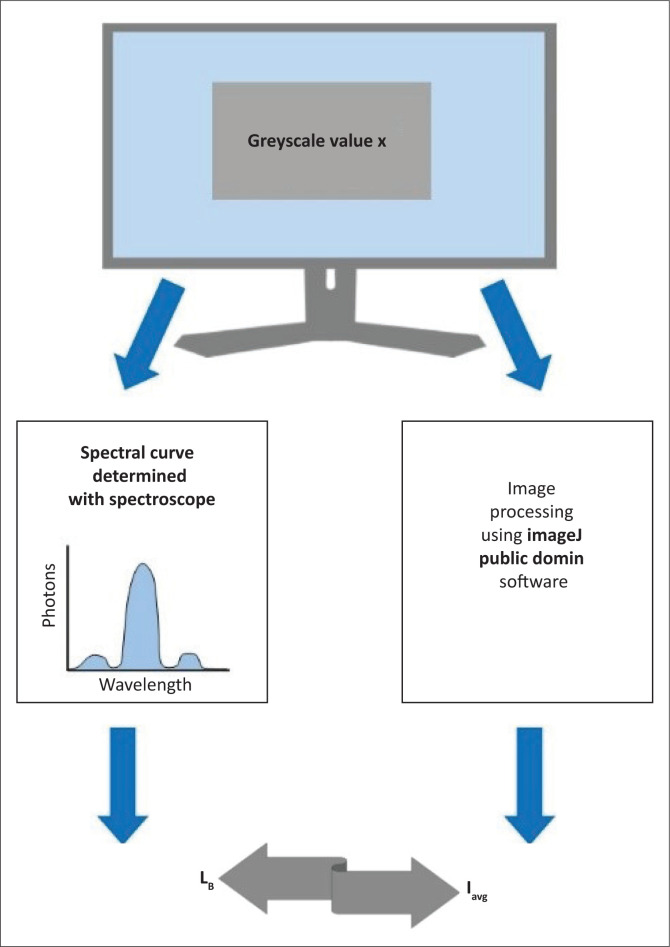
Known greyscale values were displayed on dedicated diagnostic displays, and blue-light radiance, L_B_, and average intensity, I_avg_, were determined for each. The relation between L_B_ and I_avg_ for known greyscale values provided means to deduce L_B_ values for assessed radiographic images.

As blue-light radiance for different greyscale values was known from spectral measurements, it was possible to deduce blue-light radiance for the different average image intensities of selected modalities (Figure 6).

### Ethical considerations

An application for full ethical approval was made to the Health Sciences Research Ethics Committee of the University of the Free State and ethical approval was received on 10 June 2021 (ref. no. UFS-HSD2021/0153/2906). All procedures performed in studies involving human participants were in accordance with the ethical standards of the institutional and national research committee and with the 1964 Helsinki Declaration and its later amendments or comparable ethical standards. Written informed consent was obtained from all individual participants involved in the study.

## Results

The average viewing time that registrars spent in front of DDDs on a standard workday was determined as 6 h 20 min (380 min) with a median of 06 h15 min (375 min). This included time spent reporting and reviewing images on workstation monitors and excluded times when registrars were physically away from their stations. The average viewing time of 380 min was used for further calculations (Table 2). All participants were considered senior registrars and were within 14 months from sitting for their final exit exams.

**TABLE 2:** Average daily viewing times reported from participants.

Description	Participant 1	Participant 2	Participant 3	Participant 4
Average daily viewing time	06:53:42	07:27:00	05:26:03	05:36:26

Note: Time values indicated in minutes for ease of interpretation.

Effective blue-light radiance, *L_B_*, was determined for a spectrum of different greyscale values according to Equation 1. Summation of measured spectral values and weighting with the blue-light hazard function to encompass sensitivity of the normal and aphakic human eye was performed. A paired-sample *t*-test was conducted to compare effective blue-light radiance, *L_B_*, with hazard function weighting for normal and aphakic eyes applied. There was no significant difference in the results of effective blue-light radiance, *L_B_*, between using normal eye hazard function (mean = 0.137, standard deviation [s.d.] = 0.117) and aphakic hazard function (mean = 0.137, s.d. = 0.118); *t*(13) = −0.081, *p* = 0.936. Only normal hazard function weighting was thus used. Using the relation of average image intensity (*I_avg_*) to a specific greyscale value, the effective blue-light radiance, *L_B_*, was determined for CXR, CT brain and MRI brain (Table 3).

**TABLE 3:** Effective blue-light radiance values determined for average intensity values of different imaging modalities.

Average intensity (*I_avg_*)/greyscale	Value	Range	Effective blue-light radiance (W/cm^2^sr)
Chest radiograph	56	106	0.022 × 10^−6^
CT brain	38	33	0.019 × 10^−6^
MRI brain	32	15	0.016 × 10^−6^

**FIGURE 7:**
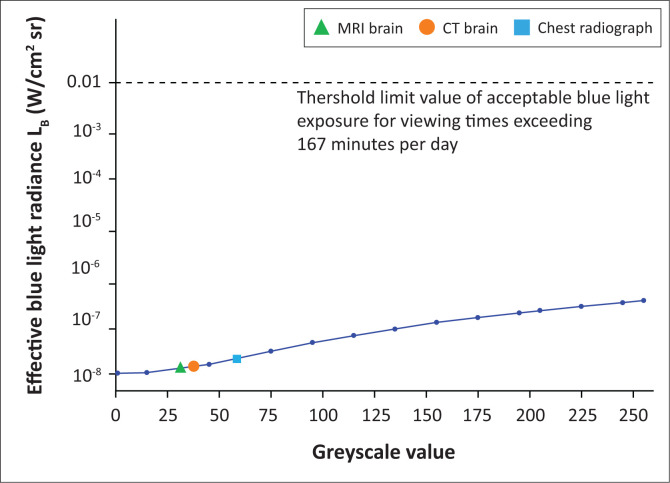
Effective blue-light radiance, L_B_, as determined for each greyscale value ranging from 0 to 255. Average image intensities for MRI and CT brain and CXR indicated, with effective blue-light radiance between 0.01 W/cm^2^sr and 0.022 W/cm^2^sr, which is far below the recommended threshold limit value.

Average image intensities were determined as 56 for CXR, 38 for CT brain and 32 for MRI brain. These values correspond to 56, 38 and 32 on the greyscale, respectively. For visual clarity, the authors represent this on a logarithmic scale, as the measured results were very small and the difference between the threshold and measured values was substantial (Figure 7).

According to ICNIRP and ACGIH^®^ guidelines, as the average daily viewing time of registrars exceeds 167 min, recommended acceptable exposure falls in category 3 in Table 1. According to Equation 2, effective blue-light radiance in this group should be below 0.01 W/cm^2^sr.^16,17^ A one sample *t*-test was performed to compare the mean effective blue-light radiance, *L_B_*, to the threshold limit of acceptable blue-light exposure at viewing times exceeding 167 min per day. The mean value of effective blue-light radiance, *L_B_*, (mean = 0.137, s.d. = 0.118) was significantly different from the threshold limit of acceptable blue-light exposure at viewing times exceeding 167 min per day; *t*(13) = −318216.031, *p* < 0.001. It was clear that the determined blue-light radiance values were drastically lower than the recommended threshold limit value represented by the dotted line (Figure 7). Daily blue-light exposure in terms of blue-light radiance was concluded to be more than 10 000 times below the maximum limit.

## Discussion

According to the CIE, light sources with luminance of less than 10 000 cd/m^2^ are not likely to exceed exposure limits.^24^ The monitors used for reporting at the institution under study all have a luminance of 500 cd/m^2^, which is markedly less than the values that the CIE deem significant. The authors’ concern was that because of the prolonged time radiologists spend in front of these monitors, the radiance may be increased and exceed the recommended threshold values.^25^

Unlike the well-defined deterministic dose ranges for ionising radiation dose effects, specific phototoxic retinal dosages have, according to the authors’ knowledge, mainly been described in animal models and *in vitro* studies thus far.^15,26,27^ Exact values for non-ionising visible light dosages in terms of specific phototoxic effect, chronicity of exposure and possibility of subclinical disease in the human retina remain ambiguous.^28^ The ACGIH^®^ and ICNIRP recommends exposure values in terms of ranges of acceptable effective blue-light radiance and maximum dose approximations, but exact dosages remain vague.

The retinal dosage of blue light is determined by many factors, including properties of the eye itself, physiological and health status of the viewer and external influences. Pupil diameter, focal length, eye movements and characteristics of the structures comprising the eye regulate the area of retina that is exposed.^17,29^ Pupillary response is highly variable between individuals. Age is an important parameter that determines how responsive an individual’s pupil reaction is. Corneal thickness was found to be the main morphological determinant of pupillary response.^30^ Noise levels, temperature, individual medication use and consumption of caffeine and other stimulants are also known to influence pupillary response.^29^ Underlying medical conditions such as diabetes, glaucoma and neuropsychiatric disease can influence pupillary responsiveness and ultimately retinal blue-light dosage.^29,31,32^ Ambient lighting plays a dual role in blue-light exposure. The lighting conditions will determine the level of pupillary dilatation, which will affect retinal dosage.^33^ Consequently, one can expect a variety of individual experiences when it comes to the eventual retinal energy deposition from emitted blue light.

Effectively, eye movement will enlarge the area of the retina upon which blue light photons are incident. In case of prolonged viewing with decreased eye movements, such as intense evaluation of an image, the blue light dosage to the retina will thus be higher.^17^ General lighting may add to blue-light radiance because of the blue-light components in LED and other incandescent light sources. Previous studies, however, proved that blue-light exposure originating from light sources is unlikely to exceed any threshold limits.^34^ The authors only assessed direct DDD unit emission and did not include additional blue light from sources such as glare from other monitors, incandescent or fluorescent luminaires and mobile devices. When viewing DDDs, the same optimal viewing distances and angles are not always used. It is advised that the ideal viewing distance should be approximately 60 cm, at a viewing angle directed slightly downward.^35^ In practice, these conditions are not always strictly adhered to, causing differences in the blue-light radiance that ultimately reach the retina. Cognitive factors such as the content being viewed and the associated emotions that an individual links to it are postulated to also contribute to pupillary response.^36^ For simplification, the effect of these external factors was not considered in this study. An experimental setup to accurately mimic retinal blue-light deposition will be technically and logistically near impossible; hence, threshold limits, time ranges and estimates were used in this study.

This assessment of blue-light exposure in registrars yielded effective blue-light radiance values of more than 10 000 times below the recommended safe value of 0.1 W/cm^2^sr. This is even more striking when comparing the measured results to other well-known sources of blue light. Bullough et al. demonstrated blue-light radiance from the sun in less than 1 s as 1.2 × 10^6^ 0.1 W/cm^2^sr. A standard fluorescent light source (8 RE 4100 K) yielded a blue-light radiance of 5.6 W/cm^2^sr.^37^ Blue-light radiance from DDDs in a radiological setting is not only much lower than threshold values but also markedly less than the blue-light exposure an individual would receive from the sun or a standard fluorescent light source.

Limitations of this study include the small study population, self-reported viewing times and the exclusion of the multitude of variables that may influence retinal blue-light deposition as described.The viewing time determined by the authors was carried out using a basic method of activating a stopwatch while reporting. Independent verification of viewing times by means of an objective observer measuring time or assessment of login time data from PACS was not performed. Although all registrars were dedicated and actively participating in the process, it should be considered that viewing times may have been either over- or underestimated because of human error.

The study’s evaluation was carried out using three common imaging modalities, that is, CXR, CT brain and MRI brain. These modalities are often the workhorses in radiology, but a wide variety of other types of images are also read. The brightness of the viewed content will determine blue-light exposure; hence, brighter-appearing images such as fluoroscopic studies will most likely yield more blue light than a darker mammography study. Other modalities were omitted for the sake of simplicity and reproducibility of the study.

The DDD units at the institution under study are compliant with local regulatory standards, but each device remains unique. As a result of technical variances, different display units may, within limits, vary in their emission of blue light. It is also important to consider that DDD units are not the only devices emitting blue light that radiology practitioners are exposed to. Often, practitioners will review images at machine terminals immediately after acquisition to decide whether further images or sequences are required. These monitors also emit blue light but were not included in our measurements. Furthermore, in the current era of electronic device usage for a multitude professional and personal duties, a large component of blue-light exposure that will add to retinal dosage is assumed to come from these devices. Although beyond the scope of this study, this method can be used in future research to quantify blue light from, for instance, cellular phones, tablets, personal computers and televisions. Based on the current study’s results and the CIE position on sources with a luminance of less than 10 000 cd/m^2^, however, the authors do not expect the component of blue-light exposure from above-mentioned devices to be significant at all.^24^

Although measured values were well below threshold limits, a clear relation between the viewed content and blue-light radiance was determined. For darker images with lower average intensities such as MRI, less blue-light radiance was determined than for brighter chest radiographs. Thus, if brighter images with increased content in the upper ranges of the greyscale are viewed, more effective blue-light radiance will be measured. However, as evident from the higher greyscale values in Figure 7, if an individual was viewing even maximal greyscale value content (i.e. a white screen) for the duration of one working day, blue light radiance would still be well within the ranges considered safe.

Recent studies were not able to establish clear evidence to advocate for the use of glasses with blue light–blocking lenses. Available research on blue-blocking lenses is mainly based on animal data and laboratory-centred experiments rather than extensive human clinical trials, leaving potential paucities in clinical applications.^38,39^ A 2021 study performed by Singh et al. established no change in digital eye strain when using blue light–blocking glasses among 120 study participants. Moreover, the blue-light hazard in typical environments is being questioned recently as a speculative concept used to generate blue light–phobia among consumers and in clinical practice settings.^40^ This study’s findings support this notion. The authors established that the amount of blue light emitted from diagnostic workstation monitors to which participants in this study were exposed on a daily basis was well below the levels that are regarded as occupationally safe.

Future research in a study population consisting of a wider variety of practising radiologists, including the public and private sectors, may yield sensible information regarding the role that different work circumstances play. Other imaging modalities can be assessed to provide a more exhaustive overview of blue-light exposure. Independent observation of viewing time by means of computer login data or motion sensors can be considered to reduce human error that may occur with self-measured viewing times. With minor modifications, the methods and results of this study can essentially be extrapolated to any industry requiring employees to work on a VDU and where there is concern regarding occupational blue-light exposure.

## Conclusion

The dangers of blue light and its effect on ocular and general health are well documented. The scope of practice requires healthcare practitioners in diagnostic radiology to spend long hours in front of DDD units that use LED technology, which emit high amounts of blue light. The authors’ concern was that this may predispose radiology practitioners to an occupational hazard related to blue light and its sequelae. It was found that the average time a radiology registrar spends in front of a monitor per workday is 380 min. The content that was being viewed played a part in the blue light exposure. Brighter chest radiographs demonstrated more blue-light radiance than darker MRI brain images. Nonetheless, exposure to effective blue-light radiance from monitors during a standard working day was far below the ranges that are recognised as hazardous.
